# Comparing perioperative outcomes after transmetatarsal amputation in patients with or without peripheral vascular disease

**DOI:** 10.1002/jfa2.70026

**Published:** 2025-02-09

**Authors:** Mark A. Plantz, Rachel Bergman, Erik Gerlach, Muhammad Mutawakkil, Milap Patel, Anish R. Kadakia

**Affiliations:** ^1^ Department of Orthopaedic Surgery Northwestern University Chicago Illinois USA

**Keywords:** chronic foot wound, diabetes mellitus, perioperative complications, peripheral vascular disease, transmetatarsal amputation

## Abstract

**Background:**

Transmetatarsal amputation (TMA) is a commonly performed procedure for gangrene in the setting of diabetes or peripheral vascular disease. The purpose of this study is to investigate the incidence of and risk factors for reoperation and perioperative complications after TMA in patients undergoing surgery for primarily infectious/diabetic wounds versus peripheral vascular disease.

**Methods:**

Patients undergoing TMA between January 1, 2015 and December 31, 2020 were identified using the American College of Surgeons National Surgical Quality Improvement Program database. The indication for surgery was reported using the International Classification of Disease 9/10 codes. Patients were categorized into two groups: patients undergoing surgery for primarily infectious/diabetic wounds versus peripheral vascular disease. The incidence of 30‐day mortality, readmission, reoperation, nonhome discharge, and various medical and surgical complications was reported. Outcome measures were compared between the diabetic and peripheral vascular disease groups. Logistic regression was used to identify independent risk factors for each outcome measure of interest.

**Results:**

3392 patients were included in the final cohort. There was a 30‐day mortality rate of 2.9%, reoperation rate of 13.8%, readmission rate of 16.8%, surgical complication rate of 22.2%, and medical complication rate of 15.8%. Patients undergoing surgery for a vascular indication had a higher rate of mortality, reoperation, hospital readmission, nonhome discharge, and various medical complications (*p* < 0.05). Patients undergoing surgery for infectious/diabetic wounds had a higher rate of deep surgical site infection and systemic sepsis (*p* < 0.05). A vascular surgical indication was independently associated with reoperation and overall medical complications (*p* < 0.05). Various factors, including age, body mass index, medical comorbidities, and the presence of preoperative sepsis were associated with poor outcomes.

**Conclusion:**

Significant rates of mortality, reoperation, and hospital readmission were reported after TMA. The presence of peripheral vascular disease was independently associated with reoperation and medical complications. Patients undergoing TMA, particularly for peripheral vascular disease, should be counseled about perioperative risks and indicated for surgery carefully.

## 1 INTRODUCTION

Transmetatarsal amputation (TMA) remains a commonly utilized procedure for management of gangrene and infection in the setting of diabetes and peripheral vascular disease. Compared to below knee amputation, it provides a less complex procedure, less blood loss, and preservation of the ankle joint [13, 15]. Furthermore, TMA can serve as a treatment modality for limb salvage, potentially increasing the chance of eventual ambulation in the appropriate setting [13, 15]. Nevertheless, there is significant morbidity after TMA, particularly in patients with underlying medical comorbidities. Several studies have demonstrated a high rate of surgical complications and overall morbidity after these procedures [2, 14, 20].

Wound healing complications, recurrent infection, and dehiscence can necessitate hospital readmission, revision procedures, delayed ambulation, and a significant burden of morbidity for patients [9, 15, 22]. Conversion rates to higher levels of amputation, including below or above knee amputation, are significant [5]. Patient selection, surgical indication, and amputation level selection is crucial to minimize complications and maximize function, particularly in patients with complex medical conditions such as diabetes mellitus and peripheral vascular disease.

Despite the frequency in which TMA procedures are performed and the significant risk of postoperative complications, there is a lack of large population studies investigating complications after TMA for different surgical indications. The purpose of this study is to utilize a validated national surgical database to investigate the incidence of and risk factors for short‐term complications, namely reoperation, after TMA in patients with or without peripheral arterial disease.

## 2 MATERIALS AND METHODS

### 2.1 Data source

All data were extracted from the American College of Surgeons National Surgical Quality Improvement Program (ACS NSQIP) surgical database [1]. ACS NSQIP reports over 150 variables, including morbidity and mortality outcome measures, after commonly performed outpatient and inpatient procedures from participating institutions [1]. Operating room logs are audited to ensure correct sampling of cases, and the results of these audits have yielded an overall disagreement rate of about 2% for all variables [1]. The database has been utilized in numerous studies investigating surgical outcomes [7, 10, 16, 17].

### 2.2 Data extraction

The ACS NSQIP database was queried to identify patients undergoing TMA between January 1, 2015 and December 31, 2020 for all indications using Current Procedural Terminology (CPT) code 28805–Amputation Foot, Transmetatarsal. The International Classification of Disease 9 and 10 (ICD‐9, ICD‐10) codes were then independently screened to identify the specific surgical indication for each case. All revision procedures, traumatic indications, and cases with concurrent procedures were excluded from the analysis.

Demographic and patient‐specific variables of interested were reported, including sex, age, body mass index (BMI), medical comorbidities, and American Society of Anesthesiologist (ASA) classification. Surgical variables of interest included surgical indication and whether sepsis or septic shock were present preoperatively. Surgical indications were grouped into infectious wounds (including diabetic foot wounds), peripheral vascular disease, and tumor/other indications.

American Society of Anesthesiologist classes were defined as previously described: Class I is a normal, healthy patient; Class II is a patient with mild systemic disease; Class III is a patient with severe systemic disease; Class IV is a patient with a severe systemic disease that constantly threatens life; and Class V is a moribund patient that is not expected to survive for 24 h with or without operation [18].

The cases were grouped into two major groups of interest: those undergoing surgery for primarily infectious/diabetic wounds versus peripheral vascular disease. Grouping was based on the reported ICD‐9 or ICD‐10 codes for the primary diagnosis, as reported in the database.

Thirty‐day outcome measures included mortality, reoperation, readmission, nonhome discharge, and various complications. We defined surgical complications as those clearly related directly to surgical intervention: superficial surgical site infection (SSI), deep SSI, wound infection, dehiscence, and bleeding requiring transfusion. We defined medical complications as other complications that may be indirectly related to surgery, anesthesia, inpatient hospitalization, or baseline comorbidities. These complications included septic shock, systemic sepsis, deep venous thromboembolism, myocardial infarction (MI), cardiac arrest, cerebrovascular accident, urinary tract infection (UTI), renal insufficiency, renal failure, pneumonia, pulmonary embolism, unplanned intubation, and prolonged ventilator use for more than 48 h.

### 2.3 Statistical analysis

Chi‐squared analysis was used to compare categorical variables–including patient demographics, surgical variables, and outcome measures–between (i) patients with and without a 30‐day reoperation after the index procedure and (ii) patients undergoing surgery for infectious/diabetic versus vascular indications. A series of stepwise binary logistic regressions were then utilized to identify variables that were independently associated with each dependent variable (e.g., outcome measure of interest), namely mortality, reoperation, readmission, nonhome discharge, surgical complications, and medical complications. The independent variables included all potentially clinically relevant variables: age, sex, BMI, medical comorbidities, ASA class, and surgical indication. Statistical significance was defined as *p* < 0.05. All statistical analyses were completed using International Business Machines (IBM) SPSS Version 24.0 (Armonk, NY. IBM Corp).

## 3 RESULTS

A total of 3392 cases were included in the final analysis. Table 1 summarizes patient demographics, medical comorbidities, and surgical variables across the entire cohort. The majority of patients were between 50 and 69 years of age (55.1%), with BMI between 18.5 and 29.9 (54.0%), and ASA class 3 (64.7%). Table 1 provides detailed information regarding demographics and baseline medical comorbidities of the cohort. There was an overall high prevalence of medical comorbidities, including diabetes (76.7%), smoking (27.5%), dialysis use or renal failure (21.8%), and hypertension (75.8%). Infection/diabetic wounds were the most common indication (82.9%) followed by peripheral vascular disease (16.3%) and tumor/other (0.8%). Sepsis was present in 7.0% of patients at the time of surgery. Septic shock was present in 0.9% of patients at the time of surgery. Table S1 provides a summary of the common CPT and ICD codes defined within the cohort.

**TABLE 1:** Summary of patient demographics, medical comorbidities, and surgical variables.

	*N* (%)
Sex	
Male	2442 (72.0%)
Female	950 (28.0%)
Age	
18–39 years	
40–49 years	
50–59 years	149 (4.4%)
60–69 years	423 (12.5%)
70–79 years	878 (25.9%)
80+ years	991 (29.2%)
	609 (18.0%)
	342 (10.1%)
BMI (kg/m^2^)	
Underweight (<18.5 kg/m^2^)	
Normal (18.5–24.9 kg/m^2^)	179 (5.3%)
Overweight (25.0–29.9 kg/m^2^)	855 (25.2%)
Obese class I (30.0–34.9 kg/m^2^)	976 (28.8%)
Obese class II (35.0–39.9 kg/m^2^)	754 (22.2%)
Obese class III (40.0 + kg/m^2^)	363 (10.7%)
Comorbidities	
Diabetes	265 (7.8%)
No	790 (23.3%)
Insulin dependent	1987 (58.6%)
Non‐insulin dependent	615 (18.1%)
Smoking	933 (27.5%)
COPD	261 (7.7%)
Ascites	12 (0.4%)
Congestive heart failure	223 (6.6%)
Hypertension	2570 (75.8%)
Renal failure	156 (4.6%)
Dialysis	583 (17.2%)
Cancer	22 (0.6%)
Bleeding disorder	709 (20.9%)
Chronic steroid use	200 (5.9%)
ASA class	
Class 1 (no disturbance)	4 (0.1%)
Class 2 (mild disturbance)	143 (4.2%)
Class 3 (severe disturbance)	2195 (64.7%)
Class 4+ (life threatening)	1033 (30.5%)
Class 5 (moribund)	4 (0.1%)
Not reported	13 (0.4%)
Surgical indication	
Infection/Diabetic wounds	2812 (82.9%)
Vascular	553 (16.3%)
Tumor/Other	27 (0.8%)
Sepsis present preoperatively	
Yes	236 (7.0%)
No	3156 (93.0%)
Septic shock present preoperatively	
Yes	32 (0.9%)
No	3360 (99.1%)

*Note*: This table provides a summary of the patient demographics, medical comorbidities, and surgical variables of the entire cohort. Specifically, it summarizes sex, age, body mass index, the incidence of various medical comorbidities, and ASA class. It also summarizes the percentage of patients undergoing surgery for specific surgical indications, and the presence of sepsis or septic shock at the time of surgery.

Table 2 summarizes 30‐day outcome measures across the entire cohort. There was a mortality rate of 2.9%, reoperation rate of 13.8%, hospital readmission rate of 16.8%, and nonhome discharge rate of 41.9%. The rate of overall surgical complications was 22.2%. The rate of overall medical complications was 15.8%.

**TABLE 2:** Overall 30‐day complications.

	*N* (%)
Mortality	98 (2.9%)
Reoperation	468 (13.8%)
Readmission	569 (16.8%)
Nonhome discharge	1421 (41.9%)
Surgical complications	
Overall	754 (22.2%)
Superficial surgical site infection	147 (4.3%)
Deep surgical site infection	182 (5.4%)
Wound infection	110 (3.2%)
Dehiscence	110 (3.2%)
Bleeding requiring transfusion	282 (8.3%)
Medical complications	
Overall	535 (15.8%)
Pneumonia	92 (2.7%)
Reintubation	56 (1.7%)
Failure to wean intubation	10 (0.3%)
Pulmonary embolism	39 (1.1%)
Renal insufficiency	29 (0.9%)
Renal failure	37 (1.1%)
Urinary tract infection	23 (0.7%)
Cerebrovascular accident	19 (0.6%)
Cardiac arrest	39 (1.1%)
Myocardial infarction	47 (1.4%)
Deep venous thromboembolism	23 (0.7%)
Systemic sepsis	47 (1.4%)
Septic shock	73 (2.2%)

*Note*: This table summarizes the overall incidence of 30‐day complications among the entire cohort. This includes mortality, reoperation, readmission, nonhome discharge, and various surgical and medical complications.

Table S2 summarizes a comparison of patient and surgical variables between patients with and without a 30‐day reoperation after TMA. There were 468 patients (13.8%) requiring reoperation. Patients requiring reoperation were more likely to have an abnormal BMI, ascites, renal failure, dialysis use, bleeding disorder, and an ASA class of 4 or 5. Patients requiring reoperation were also more likely to have undergone surgery for a vascular indication and to have sepsis present preoperatively.

Table S3 summarizes a comparison of patient and surgical variables between patients undergoing TMA surgery for infectious/diabetic wounds versus vascular indications. Patients undergoing surgery for peripheral vascular disease had more female patients, more patients in the 70–79 year, and 80+ year groups, more underweight patients, and less obese class I, II, and III patients. The vascular cohort also had more patients with Chronic Obstructive Pulmonary Disease, ascites, CHF, dialysis use, and chronic steroid use, and less patients with bleeding disorders. The vascular cohort had less patients with ASA Class 2 and 3, and more patients with ASA Class 4. The vascular cohort was also less likely to have sepsis present preoperatively.

**FIGURE 1:**
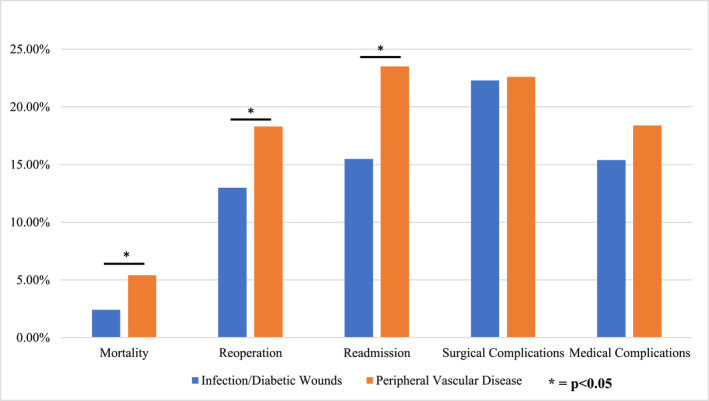
30‐day outcomes after TMA for different surgical indications. This table summarizes comparisons of the incidence of major 30‐day complications between patients undergoing surgery for infection/diabetic wounds versus vascular indications. * denotes statistical significance (*p* < 0.05).

Table 3 and Figure 1 summarize a comparison of 30‐day outcomes between patients undergoing TMA surgery for infection/diabetic wounds versus vascular indications. Patients undergoing surgery for peripheral vascular disease were more likely to experience 30‐day mortality (2.4% vs. 5.4%, *p* < 0.001), reoperation (13.0% vs. 18.3%, *p* = 0.001), hospital readmission (15.5% vs. 23.5%, *p* < 0.001), and nonhome discharge (40.3% vs. 50.8%, *p* < 0.001). Patients undergoing surgery for peripheral vascular disease were also more likely to have various medical complications, including pneumonia (2.3% vs. 4.7%, *p* = 0.002), reintubation (1.3% vs. 3.6%, *p* < 0.001), failure to wean intubation (1.0% vs. 2.0%, *p* = 0.036), UTI (0.5% vs. 1.4%, *p* = 0.017), cardiac arrest (1.0% vs. 2.2%, *p* = 0.015), and MI (1.1% vs. 3.1%, *p* < 0.001). Patients undergoing surgery for infectious/diabetic foot wounds were more likely to have a deep SSI (5.8% vs. 3.5%, *p* = 0.025) and post‐surgical systemic sepsis (9.1% vs. 6.1%, *p* = 0.025).

**TABLE 3:** Comparing 30‐day outcomes between patients undergoing TMA for infection/diabetic wound versus vascular indications.

	Infection/Diabetic wound indication [*n* = 2812]	Vascular indication [*n* = 553]	*p*
Mortality	67 (2.4%)	30 (5.4%)	**<0.001**
Reoperation	366 (13.0%)	101 (18.3%)	**0.001**
Readmission	436 (15.5%)	130 (23.5%)	**<0.001**
Nonhome discharge	1134 (40.3%)	281 (50.8%)	**<0.001**
Surgical complications			
Overall	626 (22.3%)	125 (22.6%)	0.860
Superficial surgical site infection	113 (4.0%)	32 (5.8%)	0.061
Deep surgical site infection	163 (5.8%)	19 (3.4%)	**0.025**
Wound infection	94 (3.3%)	16 (2.9%)	0.587
Dehiscence	94 (3.3%)	16 (2.9%)	0.587
Bleeding requiring transfusion	230 (8.2%)	51 (9.2%)	0.418
Medical complications			
Overall	432 (15.4%)	102 (18.4%)	0.070
Pneumonia	66 (2.3%)	26 (4.7%)	**0.002**
Reintubation	36 (1.3%)	20 (3.6%)	**<0.001**
Failure to wean intubation	27 (1.0%)	11 (2.0%)	**0.036**
Pulmonary embolism	7 (0.2%)	3 (0.5%)	0.246
Renal insufficiency	20 (0.7%)	8 (1.4%)	0.082
Renal failure	29 (1.0%)	8 (1.4%)	0.392
Urinary tract infection	15 (0.5%)	8 (1.4%)	**0.017**
Cerebrovascular accident	15 (0.5%)	4 (0.7%)	0.586
Cardiac arrest	27 (1.0%)	12 (2.2%)	**0.015**
Myocardial infarction	30 (1.1%)	17 (3.1%)	**<0.001**
Deep venous thromboembolism	18 (0.6%)	4 (0.7%)	0.824
Systemic sepsis	255 (9.1%)	34 (6.1%)	**0.025**
Septic shock	55 (2.0%)	17 (3.1%)	0.097

*Note*: This table compares the incidence of 30‐day complications between patients undergoing surgery for infection/diabetic wounds versus vascular indications. This includes mortality, reoperation, readmission, nonhome discharge, and various surgical and medical complications. The *p* values resulting from statistical analysis (i.e., Chi‐squared or Fischer’s exact tests) are also provided. Statistical significance is defined as *p* < 0.05.

Table 4 summarizes results from the multivariate logistic regression identifying independent risk factors for various 30‐day outcome measures. Advanced age, underweight BMI, CHF, diabetes, bleeding disorder, and septic shock present at the time of surgery were all independent predictors of mortality. Dialysis use, bleeding disorders, sepsis present at the time of surgery, and a vascular surgery indication were all independently associated with reoperation. Advanced age, hypertension, dialysis use, and sepsis present preoperatively were all associated with readmission. Advanced age, CHF, dialysis use, and sepsis present preoperatively were independently associated with nonhome discharge. Diabetes, dialysis use, and sepsis or septic shock present at the time of surgery were independently associated with overall surgical complications. Advanced age, hypertension, renal failure, dialysis use, chronic steroid use, bleeding disorders, and vascular surgery indication were independently associated with overall medical complications.

**TABLE 4:** Risk factors for various 30‐day complications.

	RR [95% C.I.]
Mortality	
70–79 years‐old	2.498 [1.495–4.175]
80+ years‐old	4.448 [2.576–7.681]
Underweight	3.137 [1.489–6.612]
CHF	3.442 [2.036–5.820]
Dialysis	5.664 [3.649–8.791]
Bleeding disorder	1.644 [1.055–2.560]
Septic shock present preoperatively	6.096 [2.280–16.296]
Reoperation	
Dialysis	1.858 [1.472–2.345]
Bleeding disorder	1.325 [1.053–1.666]
Vascular surgery indication	1.508 [1.032–2.202]
Sepsis present preoperatively	2.481 [1.816–3.390]
Readmission	
70–79 years‐old	1.261 [1.006–1.580]
Hypertension	1.346 [1.069–1.693]
Dialysis	1.667 [1.339–2.075]
Sepsis present preoperatively	1.684 [1.224–2.316]
Nonhome discharge	
50–59 years‐old	1.459 [1.143–1.863]
60–69 years‐old	2.279 [1.801–2.884]
70–79 years‐old	4.446 [3.439–5.746]
80+ years‐old	6.364 [4.698–8.621]
CHF	1.791 [1.331–2.408]
Dialysis	1.998 [1.649–2.422]
Sepsis present preoperatively	1.781 [1.342–2.363]
Surgical complications	
Diabetes	1.375 [1.121–1.687]
Dialysis	1.347 [1.095–1.655]
Sepsis present preoperatively	1.814 [1.364–2.412]
Septic shock present preoperatively	3.396 [1.678–6.872]
Medical complications	
70–79 years‐old	1.756 [1.295–2.381]
80+ years‐old	1.620 [1.094–2.400]
Hypertension	1.612 [1.127–2.305]
Renal failure	1.985 [1.212–3.252]
Dialysis	1.828 [1.342–2.489]
Chronic steroid use	1.858 [1.199–2.880]
Bleeding disorder	1.390 [1.040–1.858]
Vascular surgical indication	1.642 [1.036–2.604]

*Note*: This table summarizes the results of multivariate logistic regression to identify variables independently associated with the various 30‐day outcome measures of interest. Statistically significant variables were reported as the risk ratio and corresponding 95% confidence interval.

Abberviation: RR, relative risk.

## 4 DISCUSSION

TMA is commonly performed for a variety of indications, most notably for gangrene or wounds in the setting of diabetes or peripheral vascular disease. The population undergoing these procedures often has a high burden of medical comorbidities. Furthermore, these patients may have systemic signs of illness including sepsis from the underlying infection. Surgical and medical complications are common after these procedures. Despite these considerations, there is a lack of research investigating perioperative complications, medical optimization, and the risk for reoperation after TMA for different patient populations. The purpose of this study was to utilize a large national dataset to investigate perioperative complications after TMA for specific surgical indications.

### 4.1 Mortality after TMA

A 30‐day mortality rate of nearly 3% was reported in this cohort. Older age, underweight BMI, congestive heart failure, dialysis use, bleeding disorders, and septic shock being present preoperatively were all independent risk factors for 30‐day mortality. Surgical indication was not associated with mortality on regression analysis. These findings underscore the importance of medical optimization prior to surgical intervention, when possible, particularly in a population with a high degree of underlying comorbidities. There is room for improvement in the management of these patients in the perioperative period to help reduce overall mortality. However, it should also be noted that many of these procedures are done on an urgent or even emergent basis, often for serious infections, and preoperative medical optimization may not always be feasible. Nonetheless, these data provide a framework to inform patients of risks after TMA for different indications.

Pollard et al. analyzed outcomes after TMA in a cohort of 90 patients and reported two patient deaths within 30 days postoperatively [20]. The authors found that end‐stage renal disease and nonpalpable pedal pulses were independently associated with poor healing [20]. Adams et al. similarly reported on outcomes after nontraumatic TMA in a cohort of 375 patients. The authors reported that 36.3% of patients had died within 3 years postoperatively and 36.8% had required a more proximal limb amputation [2]. Only 22.1% had healed without surgical complications [2]. End‐stage renal disease was independently associated with mortality–these patients were nearly 3 times more likely to die within 3 years postoperatively [2]. Hill et al. compared the estimated probability of mortality after TMA compared to other commonly performed surgical procedures using a national surgical database–reporting that TMA had one of the highest risks of associated mortality within 30 days of the index procedure [11]. While multiple studies have demonstrated that end‐stage renal disease is a risk factor for mortality after TMA, our study also found that advanced age (70+ years), and underweight BMI, CHF, and bleeding disorders were also independently associated with 30‐day mortality after TMA. To our knowledge, these risk factors for mortality after TMA were not previously described. Patients with any of these risk factors should be counseled appropriately and medically optimized prior to any intervention, particularly if surgery is being done on a nonurgent basis.

### 4.2 Reoperation after TMA

The risk of reoperation after TMA in our cohort was substantial. Nearly 14% of patients underwent a reoperation within 30 days of the index surgery. Thorud et al. published a metaanalysis investigating reoperation and reamputation after TMA, reporting a reoperation rate of 26.9%, a reamputation rate of 29.7% and a major amputation rate of 33.2% of patients [22]. Jupiter et al. reported that minor amputations, such as TMA, were 2.5 times more likely to require short‐term irrigation and debridement compared with major amputations [13]. However, minor amputation patients were significantly less likely to require a blood transfusion or develop UTI after surgery [13]. Identifying the appropriate patients for TMA remains paramount to optimizing patient outcomes and minimizing excessive healthcare utilization after failed minor amputation procedures.

### 4.3 Risk factors for complications after TMA

Identifying risk factors for poor outcomes is an important step in defining appropriate surgical indications. Ammendola et al. published a systematic review investigating the use of TMA for diabetic foot gangrene and found that data regarding patient selection, specific surgical indications, and contraindications were sparse in the current literature [4]. Landry et al. assessed multiple variables as potential predictors of wound healing after TMA, including demographic characteristics, preoperative vascular status, and several perioperative variables. The authors reported a high rate of poor wound healing after TMA (14%) but could not identify any independent variables associated with this outcome [15]. Our study harnessed the statistical power of a large national database. We specifically identified that a vascular indication for surgery was an independent risk factor for short‐term reoperation after TMA. Additionally, dialysis dependence, the presence of preoperative sepsis, and bleeding disorders were independent risk factors for reoperation. The conclusions regarding any association of peripheral arterial disease with risk of reoperation or reamputation in prior studies have been conflicting, however [22]. Nguyen et al. reported that ankle‐to‐brachial index (ABI) ratios were similar between patients with failed or successful TMA procedures [19]. Others have reported that ischemia is directly associated with failed TMA [12]. Interestingly, Shi et al. reported that the timing of any vascular surgery intervention–performed either before or after TMA–was not associated with limb loss or wound healing [21]. Nonetheless, the risk of reoperation or reamputation should be considered carefully in patients undergoing TMA, particularly with underlying vascular disease.

### 4.4 Influence of renal function on outcomes after TMA

Poor renal function is also a known risk factor for reoperation after TMA [3]. Ahn et al. reported that elevated serum creatinine, blood urea nitrogen, dialysis use, and the stage of Chronic Kidney Disease (CKD) were associated with reamputation [3]. Patients with CKD stage IV‐V had markedly increased odds of reamputation after TMA [3]. A minor amputation in patients with advanced CKD is unlikely to be a viable long‐term solution. Although a higher level amputation can result in more functional impairment, unsuccessful attempts at limb salvage in patients with poor wound healing potential creates a significant burden on the patient’s health and the healthcare system itself.

### 4.5 Readmission after TMA

Hospital readmission remains an important metric for quality improvement. Unplanned hospital readmission yields a significant burden to the patient, increases the risk for hospital‐acquired infections and iatrogenic injuries, and burdens the healthcare system by increasing costs and utilization. We found advanced age, hypertension, dialysis use, and the presence of sepsis preoperatively for all independent predictors for 30‐day hospital readmission in this cohort. Interestingly, patients undergoing surgery for a vascular indication were not at higher risk of unplanned hospital readmission. Similar to our findings, Casciato et al. recently reported that geriatric patients were at a significantly increased risk of unplanned hospital readmission after outpatient TMA [8]. However, they did not find that any other patient demographic variables, past medical history, or surgical presentation were associated with readmission [8]. Beaulieu et al. reported on the incidence and predictors of readmission after minor lower extremity amputations more generally in the vascular surgery population; they found that elective admission, peripheral arterial disease, and chronic renal insufficiency were associated with readmission [6]. Furthermore, reamputation occurred in 95% of patients readmitted to the hospital, and 64% underwent major limb amputation (below knee, through knee, or above knee amputation) [6].

### 4.6 Comparing outcomes after TMA for diabetic versus peripheral vascular disease indications

Patients undergoing TMA for infections with underlying diabetes and/or peripheral vascular disease are clearly different patient populations with unique risk factors for complication. However, there is currently a lack of research directly comparing outcomes after TMA for these two distinct patient populations. Kanter et al. previously reported that patients undergoing TMA for diabetic foot wounds had a short‐term wound complication rate of nearly 11.9%, and obesity was associated with higher wound complications in that cohort [14]. Younger et al. previously reported the elevated risk of TMA failure in patients with hemoglobin a1c (HbA1c) values greater than 8–concluding that surgery in patients with HbA1c values less than 8 should not be performed unless the indication is to save life or limb [23]. Our nonvascular cohort also had a particularly high incidence of wound complications (16.4%)–including infection and dehiscence. Notably, the deep SSI rate in the nonvascular cohort was nearly double that of the vascular cohort–5.8% versus 3.4%, respectively. Compared to the infectious/diabetic wound group, the peripheral vascular disease cohort had unique complication profiles after surgery. Shi et al. reported that patients undergoing TMA for peripheral arterial disease had an overall 44% rate of eventual limb loss, and that the time between vascular intervention and TMA had no association with wound healing or limb loss [21]. However, there is a lack of existing data comparing outcomes after TMA for patients with or without peripheral vascular disease. Notably, our cohort found that patients undergoing TMA for peripheral arterial disease were at increased risk of many short‐term complications, including mortality, reoperation, hospital readmission, nonhome discharge, and several medical complications, as described previously. The vascular surgery cohort had more than double the mortality rate compared to the diabetic cohort, 5.4% versus 2.4%, respectively. This increased risk of serious perioperative complications must be considered when electing to proceed with limb salvage operations like TMA in patients with peripheral vascular disease.

### 4.7 Limitations

There are limitations of this study that must be discussed in the context of the conclusions herein. The retrospective nature and data source of the study introduces inherent risk of selection bias. The participating institutions included in the ACS NSQIP database have a trend toward more academic, tertiary care centers, which may limit generalizability of the findings. The database also lacks specific surgical variables that may be important for determining the risk of complications, such as the overall severity of the arterial disease, the severity or acuity of the infectious wounds, and surrounding soft tissue integrity. Outcomes data are only reported for the first 30 days postoperatively, so mid‐ and long‐term outcomes cannot be assessed. Additionally, the specific indications for reoperation are not provided and therefore cannot be reported in this study.

Lastly, the authors acknowledge that there is often overlap between patients with diabetes and peripheral vascular disease. As summarized in the supplemental data, nearly 60% of patients undergoing surgical intervention for peripheral vascular disease had been diagnosed with diabetes. The reported primary ICD‐9/10 code was used to define the main indication for surgery (i.e., diabetic wounds vs. peripheral vascular disease). Multivariate logistic regression was used to help control for potential confounding variables but this limitation must be considered in the context of the results. Nonetheless, the database provides a large validated dataset with significant statistical power, which is particularly useful for assessing differences in less common complications, which smaller sample studies may not be adequately powered to detect.

## 5 CONCLUSION

TMAs are commonly performed in a high‐risk population, with a significant risk of perioperative morbidity and mortality. The risk of short‐term reoperation is substantial, particularly in patients undergoing surgery in the setting of peripheral vascular disease. Dialysis use, bleeding disorders, a vascular surgery indication, and sepsis being present preoperatively were all independently associated with 30‐day reoperation in this population. A number of risk factors associated with poor 30‐day outcome measures were identified and described herein. Understanding these risks is important when indicating patients for surgery, choosing the appropriate amputation procedure, and ensuring patients have informed consent prior to surgery. Further investigation with prospective cohorts can help expand these findings.

## AUTHOR CONTRIBUTIONS

M.A.P: Manuscript writing; manuscript editing; data/statistical analysis; conceptualization; and methodology.

R.B.: Manuscript writing; manuscript editing; and data/statistical analysis.

E.G.: Manuscript editing and methodology.

M.M.: Conceptualization; manuscript editing; and supervision.

M.P.: Conceptualization; manuscript editing; and supervision.

A.R.K.: Conceptualization; manuscript editing; supervision; and project administration.

## CONFLICT OF INTEREST STATEMENT

M.A.P: No conflicts of interest to disclose.

R.B.: No conflicts of interest to disclose.

E.G.: No conflicts of interest to disclose.

M.M.: No conflicts of interest to disclose.

M.P.: No conflicts of interest to disclose.

A.R.K.: ‐ Consulting for Arthrex, Inc.

‐ Royalty payments and inventor share from Acumed, Limited Liability Company and DePuy Orthopedics, Inc.

## ETHICS STATEMENT

The data used herein were obtained from the American College 1 Surgeons National Surgical Quality Improvement Program. The data are deidentified prior to use by the authors. The research does therefore not involve “human subjects”.

## Supporting information

Tables S1–S3

## Data Availability

The data that support the findings of this study are available on request from the corresponding authors. The data are not publicly available due to privacy or ethical restrictions.
