# Supplementary material for: Comparing perioperative outcomes after transmetatarsal amputation in patients with or without peripheral vascular disease
Source: J Foot Ankle Res. 2025 Feb 9;18(1):e70026. doi: 10.1002/jfa2.70026 (PMC11807761; doi:10.1002/jfa2.70026)
Supplement: Supplementary file 1 — Tables S1–S3 [file JFA2-18-e70026-s001.docx]

**Supplemental Table Captions:**

**Table S1. CPT and ICD codes utilized for data extraction**

Table S1 provides a summary of the common CPT and ICD codes defined within the cohort.

**Table S2. Summary of patient and surgical variables between patients with and without 30-day reoperation**

Table S2 compares patient and surgical variables between patients with and without a 30-day reoperation. The p values resulting from statistical analysis (i.e. Chi-squared or Fischer’s exact tests) are also provided. Statistical significance is defined as p<0.05.

**Table S3. Summary of patient and surgical variables between patients undergoing TMA for infection/diabetic wound versus vascular indications**

Table S3 compares patient and surgical variables between patients undergoing surgery for infection/diabetic wounds versus vascular indications. The p values resulting from statistical analysis (i.e. Chi-squared or Fischer’s exact tests) are also provided. Statistical significance is defined as p<0.05.

**Supplemental Tables:**

| **Table S1.** CPT and ICD codes utilized for data extraction | |
| --- | --- |
| **Codes** | **Definition** |
| **CPT Code**  28805  **ICD-9 Codes**  171.X, 172.X, 232.X  250.X  440.X  443.X  444.X  707.X  730.X  785.X    **ICD-10 Codes**  C40.X, C43.X, C49.X  E10.X  E11.X  I70.X  I73.X  I96.X  I99.X  M62.X  M86.X | Amputation foot, transmetatarsal  Neoplasm  Diabetes  Atherosclerosis of the extremities  Peripheral vascular disease  Arterial embolism and thrombosis of lower extremity  Ulcer of the heel, midfoot, foot  Osteomyelitis involving the ankle and foot  Gangrene  Malignant neoplasm  Type I Diabetes  Type II Diabetes  Atherosclerosis of lower extremity  Peripheral vascular disease  Gangrene  Other disorder of circulatory system  Nontraumatic ischemic infarction of lower extremity  Osteomyelitis |

| **Table S2.** Summary of patient and surgical variables between patients with and without 30-day reoperation | | | |
| --- | --- | --- | --- |
|  | **Reoperation**  **[n = 468]** | **No Reoperation**  **[n = 2,924]** | **P** |
| Sex  Male  Female  Age  18-39 years  40-49 years  50-59 years  60-69 years  70-79 years  80+ years    BMI (kg/m^2^)  Underweight  Normal  Overweight  Obese Class I  Obese Class II  Obese Class III  Comorbidities  Diabetes  No  Insulin dependent  Non-insulin dependent  Smoking  COPD  Ascites  Congestive Heart Failure  Hypertension  Renal Failure  Dialysis  Cancer  Bleeding Disorder  Chronic Steroid Use    ASA Class  Class 1 (No disturbance)  Class 2 (Mild disturbance)  Class 3 (Severe disturbance)  Class 4 (Life threatening)  Class 5 (Moribund)  Not Reported  Surgical Indication  Infection/Diabetic Wounds  Vascular  Tumor/Other  Sepsis Present Preoperatively  Yes  No  Septic Shock Present Preoperatively  Yes  No | 336 (9.9%)  132 (28.2%)  24 (5.1%)  47 (10.0%)  119 (25.4%)  145 (31.0%)  94 (20.1%)  39 (8.3%)  26 (5.6%)  146 (31.2%)  120 (25.6%)  88 (18.8%)  57 (12.2%)  31 (6.6%)  99 (21.2%)  290 (62.0%)  79 (16.9%)  140 (29.9%)  36 (7.7%)  5 (1.1%)  33 (7.1%)  370 (79.1%)  35 (7.5%)  127 (27.1%)  1 (0.2%)  128 (27.4%)  28 (6.0%)  0 (0.0%)  12 (2.6%)  264 (56.4%)  189 (40.4%)  2 (0.4%)  1 (0.2%)  366 (10.8%)  452 (13.3%)  1 (0.03%)  63 (13.5%)  405 (86.5%)  8 (1.7%)  460 (98.3%) | 2,106 (72.0%)  818 (28.0%)  125 (4.3%)  376 (12.9%)  759 (26.0%)  846 (28.9%)  515 (17.6%)  303 (10.4%)  153 (5.2%)  709 (24.2%)  856 (29.3%)  666 (22.8%)  306 (10.5%)  234 (8.0%)  691 (23.6%)  1,697 (58.0%)  536 (18.3%)  793 (27.1%)  225 (7.7%)  7 (0.2%)  190 (6.5%)  2,200 (75.2%)  121 (4.1%)  456 (15.6%)  21 (0.7%)  581 (19.9%)  172 (5.9%)  4 (0.1%)  131 (4.5%)  1,931 (66.0%)  844 (28.9%)  2 (0.1%)  12 (0.4%)  2,446 (72.1%)  101 (3.0%)  26 (0.8%)  173 (5.9%)  2,751 (94.1%)  24 (0.8%)  2,900 (99.2%) | 0.918  0.403  0.087  0.808  0.365  0.196  0.176  0.772  **0.001**  0.107  0.055  0.265  0.302  0.239  0.109  0.572  0.209  0.998  **0.005**  0.654  0.073  **0.001**  **<0.001**  0.207  **<0.001**  0.932  –  0.055  **<0.001**  **<0.001**  **0.036**  0.522  **<0.001**  **<0.001**  **0.012**  **<0.001**  0.065 |

| **Table S3.** Summary of patient and surgical variables between patients undergoing TMA for infection/diabetic wound versus vascular indications | | | |
| --- | --- | --- | --- |
|  | **Infection/Diabetic Wound Indication**  **[n=2,812]** | **Vascular Indication**  **[n=553]** | **P** |
| Sex  Male  Female  Age  18-39 years  40-49 years  50-59 years  60-69 years  70-79 years  80+ years    BMI (kg/m^2^)  Underweight  Normal  Overweight  Obese Class I  Obese Class II  Obese Class III  Comorbidities  Diabetes  No  Insulin dependent  Non-insulin dependent  Smoking  COPD  Ascites  Congestive Heart Failure  Hypertension  Renal Failure  Dialysis  Cancer  Bleeding Disorder  Chronic Steroid Use    ASA Class  Class 1 (No disturbance)  Class 2 (Mild disturbance)  Class 3 (Severe disturbance)  Class 4 (Life threatening)  Class 5 (Moribund)  Not Reported  Sepsis Present Preoperatively  Yes  No  Septic Shock Present Preoperatively  Yes  No | 2059 (73.2%)  753 (26.8%)  131 (4.7%)  395 (14.0%)  745 (26.5%)  824 (29.3%)  478 (17.0%)  239 (8.5%)  124 (4.4%)  651 (23.2%)  809 (28.8%)  668 (23.8%)  321 (11.4%)  239 (8.5%)  550 (19.6%)  1733 (61.6%)  529 (18.8%)  767 (27.3%)  193 (6.9%)  5 (0.2%)  174 (6.2%)  2118 (75.3%)  123 (4.4%)  428 (15.2%)  15 (0.5%)  542 (19.3%)  151 (5.4%)  3 (0.1%)  128 (4.6%)  1856 (66.0%)  811 (28.8%)  2 (0.1%)  12 (0.4%)  211 (7.5%)  2601 (92.5%)  29 (1.0%)  2783 (99.0%) | 366 (66.2%)  187 (33.8%)  15 (2.7%)  25 (4.5%)  126 (22.8%)  161 (29.1%)  126 (22.8%)  100 (18.1%)  53 (9.6%)  196 (35.4%)  160 (28.9%)  79 (14.3%)  40 (7.2%)  25 (4.5%)  224 (40.5%)  246 (44.5%)  83 (15.0%)  158 (28.6%)  66 (11.9%)  7 (1.3%)  47 (8.5%)  435 (78.7%)  33 (6.0%)  154 (27.8%)  6 (1.1%)  48 (8.7%)  166 (30.0%)  0 (0.0%)  9 (1.6%)  326 (59.0%)  215 (38.9%)  2 (0.4%)  1 (0.2%)  25 (4.5%)  528 (95.5%)  3 (0.5%)  550 (99.5%) | **<0.001**  **0.040**  **<0.001**  0.069  0.929  **0.001**  **<0.001**  **<0.001**  **<0.001**  0.938  **<0.001**  **0.004**  **0.001**  **<0.001**  **<0.001**  **<0.001**  0.533  **<0.001**  **<0.001**  **0.045**  0.093  0.103  **<0.001**  0.132  **<0.001**  **0.003**  –  **0.001**  **0.001**  **<0.001**  0.070  0.394  **0.012**  0.279 |
